# Sowing the Seeds of a Pandemic? Mammalian Pathogenicity and Transmissibility of H1 Variant Influenza Viruses from the Swine Reservoir

**DOI:** 10.3390/tropicalmed4010041

**Published:** 2019-02-27

**Authors:** Joanna A. Pulit-Penaloza, Jessica A. Belser, Terrence M. Tumpey, Taronna R. Maines

**Affiliations:** Influenza Division, National Center for Immunization and Respiratory Diseases, Centers for Disease Control and Prevention, Atlanta, GA 30329, USA; jax6@cdc.gov (J.A.B.); tft9@cdc.gov (T.M.T.); zay9@cdc.gov (T.R.M.)

**Keywords:** influenza, pandemic, variant, swine, ferret, mouse

## Abstract

Emergence of genetically and antigenically diverse strains of influenza to which the human population has no or limited immunity necessitates continuous risk assessments to determine the likelihood of these viruses acquiring adaptations that facilitate sustained human-to-human transmission. As the North American swine H1 virus population has diversified over the last century by means of both antigenic drift and shift, in vivo assessments to study multifactorial traits like mammalian pathogenicity and transmissibility of these emerging influenza viruses are critical. In this review, we examine genetic, molecular, and pathogenicity and transmissibility data from a panel of contemporary North American H1 subtype swine-origin viruses isolated from humans, as compared to H1N1 seasonal and pandemic viruses, including the reconstructed 1918 virus. We present side-by-side analyses of experiments performed in the mouse and ferret models using consistent experimental protocols to facilitate enhanced interpretation of in vivo data. Contextualizing these analyses in a broader context permits a greater appreciation of the role that in vivo risk assessment experiments play in pandemic preparedness. Collectively, we find that despite strain-specific heterogeneity among swine-origin H1 viruses, contemporary swine viruses isolated from humans possess many attributes shared by prior pandemic strains, warranting heightened surveillance and evaluation of these zoonotic viruses.

## 1. Introduction

Influenza A virus is an enveloped, negative-sense RNA virus containing a segmented genome comprising eight gene segments [1]. While aquatic birds represent the main natural reservoir, they are not the only species in which influenza A viruses have established stable lineages. Several mammalian hosts including, but not limited to, humans, pigs, horses, and dogs maintain genetically and antigenically distinct lineages of influenza viruses [2,3]. The first swine influenza viruses were isolated in the 1930s [4], representative of the classical swine H1 lineage derived from the ancestors of the 1918 pandemic virus [5]. These viruses were dominant in swine until the emergence of H3N2 viruses in pigs in the late 1990s. Phylogenetic analyses revealed that the new strains resulted from reassortment among swine, human, and avian viruses and called attention to pigs as a “mixing vessel” for different influenza viruses, potentially due to the presence of both human-like and avian-like sialic acids (SA) on the swine respiratory tract epithelium [6]. The subsequent emergence of quadruple reassortant 2009 pandemic H1N1 viruses containing two genes derived from Eurasian swine viruses further supported the role of swine in the generation of new strains with pandemic potential. Ultimately, increased antigenic drift and shift have led to a tremendous increase in diversity of viruses that currently circulate in swine [7].

While the relative role of swine as a “mixing vessel” and a source of viruses with increased fitness to humans is open to debate [8], spillovers of novel influenza viruses from any species, including swine, represent a substantial public health threat. Zoonotic influenza viruses are typically antigenically distinct from viruses that circulate in people, and therefore, are not covered by seasonal vaccinations [9]. Moreover, each zoonotic infection presents an opportunity for the virus to acquire adaptations facilitating efficient transmission among people [6,10]. For these reasons, continuous surveillance of influenza viruses from different reservoirs, and swift assessment of zoonotic-origin influenza viruses isolated from humans, are critical activities for pandemic preparedness. Several risk assessment tools have been established to improve pandemic preparedness efforts, notably the Influenza Risk Assessment Tool (IRAT) [11] and Tool for Influenza Pandemic Risk Assessment (TIPRA) [12]. These rubrics consider properties of the virus, host, and ecological factors; some of the properties in these assessments, especially multifactorial traits such as pathogenicity and transmissibility, are informed by data generated in laboratory mammalian models. As these rubrics take into account both new and historical data, there is a need to contextualize and study in-depth contemporary emerging influenza viruses alongside historical viruses that were associated with human disease. These inclusive analyses may ultimately facilitate identification of phenotypic changes and relevant molecular markers which confer mammalian virulence and transmissibility. However, given the high degree of variability in procedures and methodologies among different institutions, particularly with regard to the ferret model, it can be challenging to draw meaningful conclusions from assessments conducted in different laboratories [13].

While the majority of documented zoonotic infections have occurred following exposure to infected poultry, influenza virus-infected swine, nonetheless, represent a substantial public health threat. Swine-origin viruses that are isolated from human cases are uniquely referred to as variant viruses and are denoted by adding the letter “v” to the virus subtype [14]. Unlike seasonal epidemics which occur annually during fall and winter months in the Northern Hemisphere, variant influenza virus cases are most frequently detected during summer and fall months concurrent with agricultural fairs [15]. Three swine-origin virus subtypes have been isolated from humans to date: H1N1v, H1N2v, and H3N2v. According to the CDC, since 2010, H1 subtype viruses from the North American swine reservoir have resulted in 34 human cases with 1 fatality [15]. To better contextualize these novel viruses that pose a threat to human health, we performed a comparative analysis of human seasonal and pandemic H1N1 and swine-origin H1N1v and H1N2v influenza viruses, spanning from the reconstructed 1918 pandemic strain to contemporary viruses causing human infections today. Analyses of data generated in vivo were restricted to North American human isolates studied under uniform laboratory conditions to minimize experimental variability and facilitate comparison of virological properties. Here we examine genetic differences between selected H1 virus strains, their variable pathogenicity in the mouse and ferret models, transmissibility in the ferret model, and key molecular determinants of virulence known to influence these properties. As the tremendous genetic heterogeneity among North American H1 swine influenza viruses makes it difficult to contextualize novel influenza strains with previously studied H1 subtype viruses, this review provides an avenue to study dynamic properties in a setting that is as controlled as possible. 

## 2. Genetic Diversity of North American H1 Variant Influenza Viruses 

Evolution of viruses in the swine reservoir has been described in detail previously [7,16]. With respect to H1 subtype North American swine viruses, the human-seasonal and classical swine influenza A viruses represent distinct lineages that likely emerged prior to the 1918 H1N1 pandemic [5]. The first classical swine virus, isolated in 1930, shared genetic and antigenic similarities with the 1918 virus [17,18]. In support of this, all genes from the reconstructed 1918 virus are reminiscent of the classical swine lineage, as is the virus responsible for one of the largest clusters of swine H1 virus infections in soldiers at Fort Dix, New Jersey in 1976 (NJ/08) [19,20]. This virus had the ability to spread among people causing 230 estimated cases, 13 hospitalizations, and 1 death [21]. In contrast, human seasonal lineage influenza virus, represented in Table 1 by A/Brisbane/59/2007 (Bris/59) virus, has all genes derived from the human seasonal lineage [22]. As mentioned in the introduction, occasional coinfection of swine with avian and human influenza viruses may lead to genetic reassortment and generation of new influenza virus strains [23,24]. In the late 1990s novel H3N2 viruses possessing genes from classical swine H1N1 viruses (NP, M, and NS), North American avian influenza viruses (PB2 and PA), and human seasonal H3N2 viruses (HA, NA and PB1) emerged in pigs [25]. Establishment of H3N2 viruses and the triple reassortant internal gene (TRIG) cassette in the swine reservoir increased the genetic diversity of influenza viruses circulating in this species. Subsequently, independent introductions of human H1 subtype viruses into the swine population and continued reassortment led to the emergence of H1N1 and H1N2 viruses in pigs containing a TRIG constellation and HA and NA genes derived from classical swine H1N1 and/or human seasonal influenza viruses [16,26]. Further evolution of H1 viruses in pigs was facilitated by the introduction of Eurasian swine lineage NA and M genes, which resulted in a new strain (referred to as H1N1pdm09) that spread globally causing the first influenza pandemic of the 21st century [27,28]. The H1N1pdm09 virus displaced previously circulating seasonal H1N1 viruses (e.g. Bris/59) and continues to circulate in the human population [29]. Since the isolation of the first swine influenza virus in 1930, classical swine lineage viruses have evolved into distinct currently circulating phylogenetic clades, including α-H1, β-H1, γ-H1, and H1N1pdm09. In contrast, human seasonal lineage viruses are represented by two clusters: δ-1-H1 and δ-2-H1, with the δ-1-H1 cluster further divided into δ-1a-H1 and δ-1b-H1 clades [7].

Due to the increased virus diversity in swine mediated by antigenic shift and drift [29,30], H1v influenza viruses exhibit pronounced heterogeneity in their surface glycoproteins and internal gene constellations. As shown in Table 1, H1 subtype viruses from multiple phylogenetic clades have been associated with human infection, underscoring the challenge faced by public health officials in assessing the relative pandemic risk posed by such a diverse array of viruses. The viruses that have jumped the species barrier from swine to cause human infections in the United States have often been antigenically distinct from H1 subtype viruses that are circulating in humans and, as such, are not covered by seasonal vaccine formulations [9,22,31]. Beyond the differences in the HA, there are also differences in the subtype and origin of NA among H1v viruses. Strains included in this analysis possess either classical swine lineage N1 NA (TX/14, OH/02, OH/09, and IA/39) or human seasonal lineage N2 NA (MN/45, MN/19, and WI/71). Variant viruses isolated prior to the 2009 pandemic (TX/14 and OH/02) possess a TRIG constellation. Following the emergence of H1N1pdm09 viruses (represented by Mex/4482, CA/04, CA/07, and TX/15), which acquired NA and M from the Eurasian swine lineage, the swine H1 virus population further diversified by reassortment [32]. The majority of the representative variant viruses isolated after the 2009 pandemic described here contain a Eurasian swine lineage M, which has been previously associated with a fitness advantage in animal models [33,34]. In addition, few of the variant viruses (MN/45 and IA/39) also possess H1N1pdm09-origin PA and NP genes. As molecular determinants of mammalian virulence and transmissibility have been identified in both surface glycoprotein and internal genes, in vivo assessments of these heterogeneous variant viruses is critical, as will be described in more detail in the following sections.

## 3. Key Determinants of Mammalian Pathogenicity and Transmissibility of H1 Variant Influenza Viruses 

Although the human population possesses limited immunity to swine influenza viruses, further acquisition of adaptations allowing for efficient infection, replication, and transmission in a new host would still be necessary for viruses currently circulating in the swine reservoir to cause a pandemic in humans. There is broad interest in identifying key features associated with human adaptation; however, prediction of pandemic viruses is difficult due to the complexity of virus–host interactions. Substantial evidence shows that factors including, but not limited to, receptor binding specificity, HA activation threshold pH, polymerase complex efficiency, HA and NA balance, NA stalk length, virus morphology, and antagonism of innate immune responses (reviewed in [37,38]) play critical roles in virus adaptation. In this review, we discuss experimental data for receptor binding specificity and HA activation, as well as provide a molecular analysis of selected key markers in the polymerase genes for influenza viruses that were previously characterized in mammalian models. Nonetheless, it is important to keep in mind that virus adaptation to a new host represents a polygenic trait which requires an optimal constellation of genes, and additional parameters not discussed in detail here may also contribute to this process [38]. 

Receptor binding preference has long been recognized as a critical molecular determinant for adaptation of zoonotic influenza viruses to humans. Avian influenza viruses preferentially bind alpha 2,3-linked sialic acid (α2,3 SA) receptors while human influenza viruses preferentially bind alpha 2,6-linked sialic acid (α2,6 SA) receptors [39]. Swine influenza viruses predominantly have α2,6 SA or dual (α2,3 and α2,6 SA) receptor specificity [39,40], which is in accord with the reported similarity of SA receptor distribution in the respiratory tracts of swine and humans [41]. Studies evaluating molecular markers required for adaptation of swine influenza viruses to humans and generation of a pandemic virus have been greatly aided by a concurrent study of the reconstructed 1918 virus due to its ability to efficiently spread among humans and cause severe illness. Early work using the reconstructed 1918 virus showed that the combination of amino acid residues at the 190 and 225 positions of HA (H3 numbering) are important for binding specificity to human SA receptors and efficient transmissibility among ferrets [42,43]. Consistent with these results, all subsequent pandemics (H2N2 in 1957, H3N2 in 1968, and H1N1pdm09 in 2009) were caused by viruses capable of binding α2,6 SA [42,44,45,46]. Based on experimental data, the D190/D225 combination of amino acids is often associated with α2,6 SA binding [43,47]. For example, the 1918 pandemic, 2009 pandemic (CA/07), and human seasonal (Bris/59) viruses possessing D190/D225 were experimentally shown to have an α2,6 SA binding preference [40,42,45,46,48] (Table 2). Furthermore, the HA of these viruses contains Q226, shown previously to play a role in H1N1 virus binding to human-type receptors and transmission among mammals [49,50]. Although experimental data often supports this consensus, binding specificities of selected H1v viruses cannot always be predicted based on the sequence in these two locations, indicating that additional residues need to be taken into consideration or additional experimental methods need to be used to more precisely evaluate binding specificity.

Among several swine viruses that have crossed the species barrier, few H1v viruses (MN/45, OH/02, and IA/39) displayed a preference for α2,6 SA receptors in a resialylated red blood cell assay, similar to viruses capable of efficient transmission among humans, including 1918, H1N1pdm09, and human seasonal H1 viruses (Table 2) [53]. Although it is well documented that transmissibility requires α2,6 SA binding specificity, the loss of α2,3 SA binding specificity may not represent a requirement for some viruses [55]. In this analysis, most of the tested variant viruses displayed efficient binding to α2,6 SA, but could also bind α2,3 SA, albeit with reduced efficacy. An additional consideration for influenza viruses possessing dual binding specificity is the ability to bind to a broader repertoire of host cells. Reports suggest that early 2009 pandemic isolates possessing the D225G mutation in the HA and dual binding specificity were associated with enhanced virulence in humans [52,56,57] possibly due to more-severe lung damage resulting from more efficient infection of human alveolar type II pneumocytes enriched in α2,3 SA receptors [58]. Overall, swine and human H1 influenza viruses have overlapping receptor requirements, which may contribute, in part, to occasional human infections with swine viruses. Despite the relatively sporadic nature of these events, each exposure presents an opportunity for further adaptation of swine influenza viruses, underscoring the pandemic risk posed by influenza viruses originating from the swine reservoir.

Another consideration for a virus with pandemic potential is the pH at which the HA mediates fusion between the viral and endosomal membranes. The cleaved HA1/HA2 of an infectious influenza virus is in a high-energy, metastable state, which, following endocytosis and exposure to a low pH environment, undergoes an irreversible conformational change that causes membrane fusion. Consequently, changes to the HA due to exposure to low pH outside of the host cell inactivates the virus [59,60]. As HA stabilization prevents virus inactivation in the mildly acidic mammalian upper respiratory tract (the nasal cavity pH in humans is ≥5.3 [59,61]), there is growing evidence that the threshold pH for membrane fusion represents an important property associated with host adaptation of influenza A viruses. The pH at which HA activation occurs for influenza viruses ranges from about 4.2 to 6.2, but, in general, the activation pH is higher among swine influenza viruses (~5.6–6.9) than human influenza viruses (~5.0–5.5) [59]. Evolution leading to lower HA activation pH was observed among precursor swine H1N1 viruses (pH 5.5–6.0), early human isolates (~5.5), and the later human isolates of H1N1pdm09 viruses (5.2–5.4). Experimental data suggest that a pH ≤ 5.5 is necessary for human adaptation [62], and, thus, this parameter may be an additional indicator of viruses with heightened pandemic potential. Accordingly, the human seasonal virus (Bris/59), 1918 pandemic, and 2009 pandemic (TX/15 and CA/07) viruses all possess pH thresholds for HA activation of ≤5.5 (Table 2). In agreement with previous observations for classical swine and triple reassortant H1 viruses [62], all variant viruses evaluated in this review were found to have HA activation pH values of 5.5–5.7, which suggests that H1v viruses have not fully adapted to humans.

Internal influenza virus genes also contribute to host adaptation and are closely studied during surveillance efforts. Notably, the composition of the influenza polymerase complex has been shown to be a crucial determinant of host adaptation [37,38]. Importantly, a limiting factor of influenza virus adaptation to humans is the ability of the virus to replicate efficiently at the lower temperatures found in the upper respiratory tract of mammals [63,64]. This feature is particularly important for avian influenza viruses, which have evolved to replicate in the avian enteric tract at 40–41 °C [65,66]. The E627K or a compensatory D701N substitution in PB2 are well-documented markers associated with efficient replication in the human respiratory tract [67,68]. While the human seasonal (Bris/59), pandemic 1918, and early H1v viruses (such as NJ/08) contain K627 in the PB2 gene, following the introduction of triple reassortant viruses in the pig population, the majority of contemporary swine viruses bear an avian-origin PB2 gene and lack these substitutions. Consequently, the H1N1pdm09 and the aforementioned variant viruses isolated since 2007 feature compensatory substitutions S590/R591 (Table 2) [69]. Notably, the presence of other substitutions in PB2, including T271A, may also enhance polymerase activity through different mechanisms than those mediated by the E627K and D701N substitutions [50,70]. Coupled with other known molecular determinants of virulence and host adaptation in internal influenza virus proteins, these analyses demonstrate the need to monitor emerging swine influenza viruses for mutations which may increase replication fitness in human hosts [37,38].

## 4. Pathogenicity of H1 Variant Influenza Viruses in the Murine Model

The mouse model is frequently employed to study wild-type influenza viruses from zoonotic sources, as most animal influenza viruses replicate efficiently in the murine respiratory tract without prior host adaptation [71]. Furthermore, the availability of laboratory reagents and transgenic strains establishes the mouse as a fundamental small mammalian model for influenza virus research. Past pandemic viruses, including the reconstructed 1918 virus and H1N1pdm09 strains, replicate to high titers in the lungs of experimentally inoculated mice, and some strains can cause fatal disease [72,73,74]. In contrast, human seasonal influenza viruses typically require mouse adaptation in order to efficiently replicate and cause severe disease in this species [71]. H1v influenza viruses generally replicate efficiency but do not typically elicit severe disease [53,75,76], although strain-specific differences may be observed.

Variations in mouse source and strain, inoculum dose and volume, and laboratory-specific experimental protocols (including choice of anesthetic and sample titration method) can affect data collected in the mouse model [77,78]. In this review, the murine pathogenicity of heterogeneous North American H1v influenza viruses was compared from studies conducted under consistent experimental conditions [53,73] (Table 3). Viruses selected for comparison include the reconstructed 1918 virus and H1N1pdm09 viruses, in addition to a panel of H1v subtype viruses. Similar to what was observed for the pandemic strains, the H1N1v and H1N2v viruses examined here replicated to high titers in mouse lungs without prior adaptation. H1v viruses isolated after the 2009 pandemic (MN/45, OH/09, IA/39, MN/19, WI/71) had the ability to replicate with increased efficiency in the noses of all inoculated mice at day three post inoculation, whereas earlier variant isolates (NJ/08, OH/02) and H1N1pdm09 viruses did not. In this comparison the most virulent strain in mice was the reconstructed 1918 virus, causing high lung titers and 100% lethality while of the four representative H1N1pdm09 viruses, only one virus (CA/07) caused 75% lethality in mice following inoculation with a high challenge dose. Despite robust replication in mouse lungs, none of the contemporary H1v influenza viruses included in this analysis caused lethality at a high inoculum dose, except for the H1N1v OH/09 virus, which was isolated during a fatal human case [31]. As mentioned earlier, substitutions in the polymerase genes have been previously associated with increased virulence in mammalian models. In particular, the OH/09 virus possesses two molecular markers, I588 in PB2 and S66 in PB1-F2 (Table 2). PB2 I588 has been shown to enhance H1N1pdm09 virus virulence by increasing replication and inhibiting interferon signaling [35,79]. PB1 was previously reported to modulate virulence in the mouse model, mediated by the PB1-F2 protein, which is translated using an alternative reading frame [75,80]. Full-length (as compared with truncated) PB1-F2 proteins are typically associated with enhanced virulence in mammals, as is the presence of a substitution at position 66 (N66S) of this protein. The 1918 virus, as well as viruses isolated during the 1997 H5N1 outbreak in Hong Kong, possessed the N66S amino acid substitution and displayed robust replication in mouse lungs and increased virulence compared with isolates bearing N at this position [81]. In addition to cell- and host-specific enhancement of replication observed previously with some strains, viruses containing this substitution also caused decreased expression of the interferons, increased levels of pro-inflammatory cytokines, and increased lung immunopathology [82].

Overall, the pandemic and H1 subtype variant strains were able to replicate in mouse lungs without prior adaptation. With one exception, the variant influenza viruses examined here did not display high virulence in this species, suggesting that although H1v influenza viruses have been undergoing continuous evolution in pigs for many years, their virulence in the mouse model has largely remained unchanged. While mice are limited in their presentation of clinical signs and symptoms of infection, characterization of H1v viruses in this model can provide supportive information for subsequent evaluation of novel vaccine and antiviral formulations against pandemic and potentially pandemic H1 subtype influenza viruses [83,84]. Furthermore, although genetically distinct from North American viruses, Eurasian swine influenza viruses were reported to cause infections ranging from mild to fatal in mice [85,86]. Because of occasional reassortment events in the swine population, it is important to conduct risk assessments inclusive of viruses of all lineages in multiple mammalian models, including the mouse. The capacity for lethal infection, high lung titers, and heightened inflammatory responses following murine inoculation with the reconstructed 1918 virus underscore the value of the mouse model for screening novel viruses for strains with increased virulence in mammalian species [72,87].

## 5. Pathogenicity of H1 Variant Influenza Viruses in the Ferret Model

The high susceptibility of ferrets to influenza virus infection, pronounced clinical symptoms and signs of infection, and the ability to transmit the virus to healthy ferrets, was first described in 1933 [88]. Currently, the ferret represents the ‘gold standard’ model for the coincident study of influenza virus pathogenicity and transmissibility, and plays an indispensable role in pandemic risk assessments of newly emerging influenza viruses [13]. Close physiologic ties between human and ferret respiratory tract tissues, and comparable distributions of sialic acid receptors throughout the upper and lower respiratory tract of both species, permit infection of ferrets with human and animal influenza viruses without prior host adaptation [71]. Clinical signs and symptoms, in general, are similar to those seen in humans including fever, weight loss, sneezing, nasal discharge, and lethargy, but can vary widely depending on the inoculum strain [88,89]. Human seasonal viruses typically cause mild disease with transient weight loss, fever, sneezing, and nasal discharge; these viruses usually replicate most efficiently in the upper respiratory tract and systemic spread is not observed. Zoonotic influenza viruses, including swine H1 viruses, in most cases, replicate in both upper and lower respiratory tract tissues of ferrets. Unlike some highly pathogenic avian influenza strains, which possess the ability to spread beyond the respiratory tract [90,91,92,93], swine-origin influenza viruses are usually restricted to respiratory tract tissues, but the virus has been occasionally detected in the olfactory bulb or intestines of ferrets [53,94]. Similar to mice, lethal outcomes are possible with select strains [69,85].

While the majority of the inoculated ferrets cleared the virus by day 8–11 post inoculation and recovered, 20% of ferrets inoculated with the reconstructed 1918 virus exhibited severe weight loss, lethargy, and succumbed to infection [42,95] (Table 4). The 1918 pandemic virus replicated robustly in the upper respiratory tract of ferrets as examined in nasal wash specimens [42,95], with nasal wash titers similar to those observed for other H1 viruses assessed in this review. Furthermore, the 1918 virus replicated equally well throughout the ferret respiratory tract (Table 5). Necrotizing bronchiolitis and alveolitis with edema were observed in histological analyses of respiratory tract tissues during the acute phase of infection [42]. In contrast, seasonal human H1 influenza viruses such as Bris/59, which circulated in humans until the 2009 pandemic, caused mild illness in ferrets and transient weight loss despite high mean peak nasal wash titers. This virus replicated most efficiently in the nasal turbinates, less efficiently in the trachea, and was not detected in the lung tissue [94]. This restriction of human seasonal influenza virus to the upper respiratory tract has also been observed for other pre-2009 H1N1 seasonal influenza viruses studied in the ferret model, including A/Solomon Islands/3/2006 [96] and A/Netherlands/26/2007 [97].

Following the 2009 pandemic, human seasonal influenza viruses were displaced by H1N1pdm09 viruses, which continue to circulate in humans. The viruses isolated during the 2009 pandemic (represented here by Mex/4482, CA/04, CA/07, and TX/15 viruses) displayed comparable tissue distributions in ferrets to the 1918 pandemic virus, including efficient replication throughout the respiratory tract and in some cases, fatal disease, primarily due to excessive weight loss during the course of infection [94,97,99]. These results are in contrast to those reported for the pre-2009 pandemic H1 human seasonal viruses, but are consistent with data observed for North American and Eurasian swine viruses, which, in general, replicated efficiently throughout the respiratory tract of ferrets, infrequently resulting in severe disease and fatal outcomes [69,85]. The pathogenicities observed in ferrets infected with triple reassortant (OH/02, TX/14, MN/19) and quadruple reassortant (MN/45, OH/09, IA/39, WI/71) North American H1v viruses were similar to those seen with swine influenza isolates [69].

Overall, the ferrets inoculated with pandemic, seasonal, or H1v viruses that were included in this analysis replicated efficiently in the ferret upper respiratory tract. Peak nasal wash titers, HA clade, and gene constellations were not a predictor of disease severity or outcome in ferrets (Table 4). Weight loss in infected ferrets varied, with mild to moderate morbidity most frequently observed. Lethality was sporadically detected and none of the tested viruses resulted in more than 50% lethality in the animals. All pandemic H1 and variant influenza viruses tested replicated throughout the respiratory tract. While H1N1pdm09 viruses exhibited increased rates of virus detection in gastrointestinal tissue [94], no consistent detection of H1v viruses in extrapulmonary tissues was observed [53]. Collectively, this work shows that H1 subtype viruses associated with human infection are capable of causing a spectrum of diseases in ferrets and many variant strains can replicate throughout the ferret respiratory tract as efficiently as the pandemic strains.

## 6. Transmissibility of H1 Variant Influenza Viruses in the Ferret Model

Similar to virus pathogenicity, the transmissibility of influenza viruses represents a multifactorial trait and cannot be studied thoroughly in the absence of a living host. The ferret and guinea pig models are most frequently employed to conduct these in vivo studies [100,101], though ferrets are preferred for risk assessment purposes [13]. Virus transmissibility, inclusive of multiple transmission modes, is typically tested by co-housing an inoculated ferret in a cage with a naïve ferret. In contrast, transmission via respiratory droplets or droplet nuclei can be evaluated by housing an inoculated ferret in a cage adjacent to a cage housing a naïve ferret, which allows for air exchange through perforations in the cage walls while preventing direct or indirect contact between animals. Typically, human seasonal and pandemic influenza viruses efficiently transmit among ferrets via respiratory droplets or droplet nuclei. Conversely, most zoonotic influenza viruses do not transmit efficiently through the air but are typically able to transmit in a direct contact setting [100,102] where they may be exposed to a higher dose of virus.

The human seasonal virus, Bris/59, transmitted efficiently between all ferret pairs in the respiratory droplet transmission model [94]. The reconstructed 1918 virus was also highly transmissible via respiratory droplets or droplet nuclei, with contact ferrets typically shedding the virus in nasal wash specimens as early as days 1–3 post-contact. The ability of the 1918 virus to transmit via air was shown to be abolished by introducing two mutations (D190E, D225G) in the receptor binding site [42], highlighting the importance of a robust human-like receptor binding preference (α2,6 SA) in the efficient transmissibility of this pandemic strain. In general, H1N1pdm09 viruses transmitted efficiently in a respiratory droplet transmission model, with some variability observed between different strains and experimental protocols [31,94,97,99]. Unlike human seasonal and pandemic viruses, the transmissibility of swine-origin H1 viruses in ferrets varies greatly between strains and is difficult to predict. Barman et al. [69] showed that North American swine triple reassortant viruses isolated from pigs prior to the 2009 pandemic transmitted efficiently between co-housed ferrets, but their transmissibility in the respiratory droplet transmission model was closely tied to HA and NA lineage. Among viruses bearing a TRIG cassette, swine influenza viruses possessing human-like HA and NA transmitted most efficiently while those containing mixed or swine-like HA and NA exhibited reduced transmissibility through the air. Consistent with these observations, the representative triple reassortant viruses, TX/14 and OH/02, which contain swine-like HA and NA, did not transmit via the air. However, the transmissibility profiles of H1v viruses isolated following the 2009 pandemic differed between strains and also did not appear to be clade-dependent. The H1v virus found to be most transmissible through the air was MN/45, an H1N2v virus containing classical swine HA and human-like NA, which transmitted between all ferret pairs by day three post-contact (Table 4). Among other H1v viruses, OH/09 and IA/39 viruses (with swine-origin HA and NA) and MN/19 and WI/71 viruses (with human-like HA and NA), displayed 33–66% transmission efficiency in the respiratory droplet transmission model. Collectively, these observations show that most swine-origin H1 viruses, including newly emergent variant strains, are capable of transmission through the air in the ferret model and, with further adaptation, may acquire the ability to transmit as well as human seasonal and pandemic influenza strains. These comparisons also underscore the difficulty of studying the contribution of individual genes in conferring a transmissible phenotype (such as HA and NA) between different strains when there is pronounced genetic heterogeneity of internal gene constellations (Table 1). Due to this genetic complexity, in vivo risk assessments (inclusive of both pathogenicity and transmissibility parameters) continue to be warranted when novel viruses from the swine reservoir cause human infections.

## 7. Conclusions

The confounding unpredictability of influenza A viruses and the occasional spillover of influenza viruses from avian to mammalian species are concerning from a public health standpoint. Swine-origin influenza viruses that cross the species barrier and cause human infections pose a considerable health threat due to their distinct antigenicity, ability to cause mammalian disease, and capacity for enhanced transmission in mammals, which are all virological attributes of pandemic influenza strains [27,31,53,103]. In addition, the emergence of the 2009 pandemic virus from pigs further supports that viruses prevalent in swine represent plausible pandemic candidates and underscores the need to closely monitor these viruses. 

In vivo and in vitro evaluation of newly emerging influenza viruses represents a key component of public health risk assessments, which guide the selection of candidate vaccine viruses to aid pandemic preparedness and serve as a basis for further research by identifying gaps in knowledge [11,104]. The information obtained using rigorous comparative meta-analyses, including the replication efficiency, relative capacity for systemic spread, and transmission frequency of different virus strains, can shed light on the identification of molecular markers of virulence and transmissibility that may not be recognized while studying viruses in isolation or under different experimental protocols [105,106]. In addition, as the ferret model is indispensable for risk assessments, meta-analyses can overcome challenges inherent in ferret research studies, such as statistical limitations pertaining to small sample sizes and the outbred nature of the model [107]. However, it is important to keep in mind that the variability of established laboratory in vivo models can often prohibit meta-analyses of data generated in mammalian models from different institutions underscoring the need for standardized laboratory protocols [13,106]. 

By employing consistent laboratory methodology and experimentation parameters among all tested H1 viruses and including the reconstructed 1918 influenza virus and well-characterized human-adapted viruses as anchors, rigorous comparison of recently isolated H1v viruses that exhibit pronounced genetic heterogeneity was possible. It is clear that the human population remains vulnerable to exposure to new, often antigenically different influenza virus strains from the swine reservoir and each human exposure brings a risk of adaptation of these viruses to transmit in humans and possibly cause a pandemic. Features such as the ability to bind to human-like receptors, and capacity for high-titer replication in human cell lines, as well as in the respiratory tract of animal models, were commonly observed with H1v viruses. In addition, the studied North American swine viruses isolated from pigs or humans displayed robust transmissibility between cohoused ferrets [31,53,69,98]. Conversely, in comparison to human seasonal and pandemic influenza virus strains, in most cases, H1v viruses transmitted less efficiently via air (in the absence of direct and indirect contact) and had higher HA activation pH thresholds, indicating a lack of human adaptation. 

In addition to infections with H1N1 and H1N2 subtype viruses, swine constitute a major source of zoonotic infections with H3N2 subtype viruses. Similar to the H1v variant viruses discussed here, swine-origin H3N2 viruses isolated from humans were also shown to be capable of mammalian infection and transmissibility in the ferret model [108,109]. As influenza virus pandemics are unpredictable, it is unknown if sporadic human infection with viruses from the swine reservoir indicates an enhanced adaptation of these viruses to humans or rather represents a series of dead-end spillover events. That said, the 2009 H1N1pdm09 pandemic underscored the rapidity in which a swine-origin influenza virus could spread globally. Continued surveillance and study of viruses from the swine reservoir, both isolated from infected swine and humans, will provide additional insight and understanding into the relative risk these influenza viruses pose to humans. Comparative analysis of new and historical strains will aid in identifying viruses that possess enhanced virulence and adaptations allowing for efficient transmission among humans and ultimately will aid in pandemic preparedness efforts.

## Figures and Tables

**Table 1:** Classification and gene origin of representative H1 subtype influenza viruses.

HA Clade			Virus Name	Virus Name in this Study			Gene Origin														
U.S.		Global			Subtype		PB2		PB1		PA		HA		NP		NA		M		NS
NA		NA	A/Brevig Mission/1/1918 A/South Carolina/1/1918	1918 ^a^	H1N1																
																				
alpha		1A.1-like	A/New Jersey/08/1976	NJ/08	H1N1v																
																			
		1A.1.1	A/Minnesota/45/2016	MN/45	H1N2v						*				*				*		
beta																					
		1A.2	A/Texas/14/2008	TX/14	H1N1v																
																			
gamma		1A.3.3.3	A/Ohio/02/2007	OH/02	H1N1v																
																				
		1A.3.3.3	A/Ohio/09/2015	OH/09	H1N1v														*		
																				
		1A.3.3.3	A/Iowa/39/2015	IA/39	H1N1v						*				*				*		
																				
	H1N1pdm09	1A.3.3.2	A/Mexico/4482/2009	Mex/4482	H1N1pdm09		*		*		*		*		*		*		*		*
																				
		1A.3.3.2	A/California/04/2009	CA/04	H1N1pdm09		*		*		*		*		*		*		*		*
																				
		1A.3.3.2	A/California/07/2009	CA/07	H1N1pdm09		*		*		*		*		*		*		*		*
																				
		1A.3.3.2	A/Texas/15/2009	TX/15	H1N1pdm09		*		*		*		*		*		*		*		*
																					
delta	delta-like	1B.2	A/Brisbane/59/2007	Bris/59	H1N1																
																				
	delta-1	1B.2.2.2	A/Minnesota/19/2011	MN/19	H1N2v																
																			
		1B.2.2.1	A/Wisconsin/71/2016	WI/71	H1N2v														*		

Clade was determined using the Swine H1 Clade Classification Tool (Influenza Research Database, www.fludb.org). Gene lineage origin was obtained from previous phylogenetic analyses [22,27,31,35]. Key: blue = classical swine H1 lineage, yellow = North American avian lineage, green = human seasonal lineage, red = Eurasian avian-like swine lineage, * H1N1pdm09 virus origin. Viruses containing triple reassortant internal gene (TRIG) cassette have NP, M, and NS genes from classical swine lineage H1N1 viruses, PB2 and PA genes from North American avian lineage viruses, and a PB1 gene from human seasonal-lineage viruses. NA – not applicable, sequence predates clade classification. ^a^ Genomic RNA of the 1918 virus was obtained from archived formalin-fixed lung autopsy materials and from frozen, unfixed lung tissues from an Alaskan influenza victim buried in permafrost during the pandemic [36].

**Table 2:** Receptor binding specificity, HA activation pH, and molecular markers of H1 influenza viruses.

Virus	Binding Specificity			Fusion pH			HA ^a^				PB2							PB1-F2
	Sialic Acid	Source		pH	Source		190	225	226		271	588	590	591	627	701		66
1918	α2,6	[42,48]		5.1	[51]		D	D	Q		T	A	G	Q	K	D		S
NJ/08	α2,6/α2,3	[39,40,52]		5.5–5.6	[53]		D	G	Q		T	A	G	Q	K	D		N
MN/45	α2,6	[53]		5.5–5-6	[53]		D	G	Q		A	T	S	R	E	D		- ^b^
TX/14	α2,6/α2,3	[40,53]		5.7	[53]		D	N	Q		A	T	S	R	E	D		N
OH/02	α2,6	[40,53]		5.6	[53]		D	D	Q		A	T	S	R	E	D		N
OH/09	α2,6/α2,3	[53]		5.7	[53]		D	G	Q		A	I	S	R	E	D		S
IA/39	α2,6	[53]		5.6–5.7	[53]		D	D	Q		A	T	S	R	E	D		N
CA/07	α2,6	[45,46]		5.5	[53]		D	D	Q		A	T	S	R	E	D		-
TX/15	α2,6/α2,3	[54]		5.4–5.5	[53]		D	D	Q		A	T	S	R	E	D		-
Bris/59	α2,6	[40]		5.3–5.4	[53]		D	D	Q		A	I	G	Q	K	D		N
MN/19	α2,6/α2,3	[53]		5.6–5.7	[53]		N	D	Q		A	T	S	R	E	D		N
WI/71	α2,6/α2,3	[53]		5.7	[53]		D	D	Q		S	T	S	R	E	D		N

^a^ H3 numbering. ^b^ Protein is truncated containing either 8 (MN/45) or 11 (CA/07 and TX/15) amino acids. IA/39 contains 79 amino acids. The full-length protein contains 90 amino acids.

**Table 3:** Pathogenicity of H1 influenza viruses in mice.

Virus	Subtype	% Weight Loss ^a^	% Lethality ^b^	Mean Viral Titer log_10_ PFU/mL ± SD ^c^				Data Source
				Day 3 p.i.		Day 6 p.i.		
				Lung	Nose	Lung	Nose	
1918 ^d^	H1N1	20.2	100	7.0 ± 0.2 (3/3)	NT	NT	NT	Unpublished
NJ/08	H1N1v	2.7	0	6.0 ± 0.2 (3/3)	ND	5.9 ± 0.9 (3/3)	ND	[73]
MN/45	H1N2v	13.0	0	6.0 ± 0.5 (3/3)	2.1 ± 0.5 (3/3)	3.8 ± 0.5 (3/3)	ND	[53]
OH/02	H1N1v	14.5	0	7.0 ± 0.3 (3/3)	1.8 (1/3)	4.5 ± 0.1 (3/3)	ND	[73]
OH/09	H1N1v	23.0	80	5.9 ± 0.4 (3/3)	3.3 ± 0.1 (3/3)	5.3 ± 0.1 (3/3)	3.6 ± 0.2 (3/3)	[53]
IA/39	H1N1v	11.1	0	5.6 ± 0.2 (3/3)	2.3 ± 0.4 (3/3)	3.4 ± 0.3 (3/3)	3.0 ± 0.4 (3/3)	[53]
Mex/4482	H1N1pdm09	19.0	0	6.4 ± 0.2 (3/3)	1.5 (1/3)	6.3 ± 0.4 (3/3)	ND	[73]
CA/04	H1N1pdm09	5.3	0	5.9 ± 0.9 (3/3)	1.5 ± 0.2 (3/3)	6.2 ± 0.1 (3/3)	ND	[73]
CA/07	H1N1pdm09	19.2	75	5.4 ± 0.2 (3/3)	1.7 (1/3)	5.3 ± 0.3 (3/3)	1.3 (1/3)	[53]
TX/15	H1N1pdm09	1.5	0	5.4 ± 1.0 (3/3)	ND	4.7 ± 0.8 (3/3)	ND	[73]
MN/19	H1N2v	6.8	0	6.0 ± 0.3 (3/3)	3.3 ± 0.2 (3/3)	4.2 ± 0.4 (3/3)	3.0 ± 0.3 (3/3)	[53]
WI/71	H1N2v	8.4	0	7.3 ± 0.3 (3/3)	4.2 ± 0.1 (3/3)	4.1 ± 0.6 (3/3)	2.2 ± 0.7 (3/3)	[53]

^a^ Mean maximum percent weight loss (*n* = 3 for 1918, *n* = 4 for CA/07, *n* = 5 for all others) following inoculation with 5.0 log_10_ PFU or EID_50_. ^b^ Percent lethality (*n* = 3 for 1918, *n* = 4 for CA/07, *n* = 5 for all others) following inoculation. ^c^ Mean viral titer expressed as log_10_ PFU/mL ± SD from animals with detectable virus titer (number of positive animals is shown in parenthesis). 1918 tissue titers were tested following inoculation at 4.0 log_10_ PFU. ^d^ Reconstructed A/South Carolina/1/1918 virus [72]. NT - not tested; 1918 inoculated mice did not survived until day 6 p.i. ND - not detected.

**Table 4:** Pathogenicity and transmissibility of H1 influenza viruses in ferrets.

Virus	log_10_ PFU/mL		% Weight Loss (%) ^c^	% Lethality ^d^	DCT ^e^			RDT ^e^		Data Source
	Inoculation Dose ^a^	NW Titer ± SD ^b^			Virus Detection	Seroconversion		Virus Detection	Seroconversion	
1918 ^f^	6.0	5.6 ± 0.2	≤13.2	20	NT	NT		3/3	3/3	[42,95]
MN/45	6.0	6.7 ± 0.6	15.6	0	3/3	3/3		3/3	3/3	[53]
TX/14	6.0	6.7 ± 0.6	11.7	33	3/3	3/3		0/3	2/3	[98]
OH/02	6.0	5.2 ± 0.5	9.4	33	3/3	3/3		0/3	1/3	[98]
OH/09	5.0	6.8 ± 0.7	13.7	11	3/3	3/3		1/3	1/3	[31]
IA/39	5.0	5.2 ± 0.1	6.0	0	3/3	3/3		2/3	2/3	[31]
Mex/4482	6.0	7.7 ± 0.2	17.5	50	3/3	3/3		2/3	2/3	[94]
CA/04	6.0	6.9 ± 0.9	10.3	0	3/3	3/3		2/3	2/3	[94]
CA/07	5.0	7.1 ± 0.4	10.6	0	NT	NT		3/3	3/3	[31]
TX/15	6.0	6.8 ± 0.6	9.1	0	3/3	3/3		2/3	2/3	[94]
Bris/59	6.0	7.0 ± 0.5	4.9	0	3/3	3/3		3/3	3/3	[94]
MN/19	6.0	6.6 ± 0.4	9.4	0	3/3	3/3		1/3	1/3	[53]
WI/71	6.0	6.7 ± 0.6	13.6	0	3/3	3/3		2/3	2/3	[53]

^a^ Each ferret was intranasally inoculated with 1 mL of the specified dose of virus diluted in PBS. ^b^ Average maximum nasal wash (NW) titer of inoculated ferrets ± SD. ^c^ Average percent maximum weight loss within 10 days post inoculation. ^d^ Percent of animals euthanized during the experiment due to excessive weight loss. For 1918 virus the value was calculated based on data from the two referenced studies. ^e^ DCT: Direct Contact Transmission model, RDT: Respiratory Droplet Transmission model; number of ferrets with detectable virus in nasal washes (virus detection) or HI titers in serum (seroconversion) over the total number of ferrets. ^f^ Reconstructed A/South Carolina/1/1918 virus [72]. NT – not tested.

**Table 5:** Influenza virus detection in ferret respiratory tract tissues.

Virus	Inoculation Dose ^a^	Mean Titer (log _10_ PFU/mL or g) ± SD ^b^			Data Source
		Nasal Turbinates	Trachea	Lung	
1918 ^c^	6.0	5.3 ± 0.9 (3/3)	5.7 ± 0.5 (3/3)	5.4 ± 0.7 (3/3)	[95]
MN/45	6.0	5.3 ± 0.2 (3/3)	4.5 ± 1.0 (3/3)	3.8 ± 2.0 (3/3)	[53]
TX/14	6.0	4.7 ± 1.0 (3/3)	4.1 ± 1.9 (3/3)	6.0 ± 1.0 (3/3)	[98]
OH/02	6.0	5.0 ± 0.7 (3/3)	4.3 ± 0.4 (3/3)	4.8 ± 1.0 (3/3)	[98]
OH/09	5.0	6.1 ± 0.6 (3/3)	4.1 ± 0.6 (3/3)	2.7 ± 0.9 (2/3)	[31]
IA/39	5.0	5.1 ± 0.1 (3/3)	3.9 ± 0.3 (3/3)	4.3 ± 0.4 (3/3)	[31]
Mex/4482	6.0	5.8 ± 0.8 (3/3)	5.0 ± 0.8 (3/3)	5.3 ± 0.6 (2/3)	[94]
CA/04	6.0	4.6 ± 0.3 (3/3)	5.0 ± 1.2 (3/3)	5.8 ± 0.4 (3/3)	[94]
CA/07	5.0	4.9 ± 0.3 (3/3)	4.9 ± 0.3 (3/3)	4.4 ± 1.4 (3/3)	[31]
TX/15	6.0	4.6 ± 0.3 (3/3)	5.7 ± 0.3 (3/3)	6.0 ± 1.0 (3/3)	[94]
Bris/59	6.0	5.6 ± 0.6 (3/3)	2.6 ± 0.1 (3/3)	ND (0/3)	[94]
WI/71	6.0	4.9 ± 0.5 (3/3)	4.6 ± 0.5 (3/3)	4.1 ± 0.5 (3/3)	[53]
MN/19	6.0	4.2 ± 0.5 (3/3)	4.1 ± 1.4 (3/3)	3.7 ± 1.0 (3/3)	[53]

^a^ Each ferret was intranasally inoculated with 1 mL of the specified dose of virus (log_10_ PFU) diluted in PBS. ^b^ Mean virus titers in tissues collected on day three post inoculation ± SD. Nasal turbinate viral titers are presented as log_10_ PFU/mL and trachea and lung titers are presented as log_10_ PFU/g of tissue. Number of ferrets with detectable titers are indicated in parenthesis. ^c^ Reconstructed A/South Carolina/1/1918 virus [72]. ND- not detected.
